# “When I Said I Wanted to Die at Home I Didn’t Mean a Nursing Home”: Care Trajectories at the End of Life

**DOI:** 10.1093/geroni/igx011

**Published:** 2017-08-30

**Authors:** Margaret Penning, Denise S Cloutier, Kim Nuernberger, Deanne Taylor

**Affiliations:** 1 Department of Sociology,; 2 Institute on Aging & Lifelong Health (IALH), and; 3 Department of Geography, University of Victoria, British Columbia, Canada.; 4 Fraser Health Authority, Surrey, British Columbia, Canada.

**Keywords:** End of life, Home and community-based care and services, Long-term care, Nursing homes, Palliative care

## Abstract

**Background and Objectives:**

Little is known regarding the care trajectories older adults experience at the end of life (EOL). We drew on a structural/institutional life course perspective to examine the trajectories evident among older adults transitioning through the Canadian formal long-term care system. The sequence of care transitions as well as the impact of social location, social and economic resources, and health-related factors on these trajectories were examined.

**Research Design and Methods:**

To identify EOL care trajectories, we used administrative data collected on older adults (aged 65+) who received publicly subsidized long-term care services (e.g., nursing home and home and community-based care) in one health region in British Columbia, Canada from January 1, 2008 through December 31, 2011 and who died by March 31, 2012 (*n* = 11,816). Multinomial logistic regression analyses assessed the impact of selected covariates on these trajectories.

**Results:**

The majority of those studied (65.4%) died outside of acute hospital settings. The most common trajectories involved transitions from home care to nursing home/residential care to non-hospital death (39.5%) and transitions from in-home care to hospital death (22.4%). These and other trajectories were shaped by social structural factors, access to social and economic resources, as well as health status and prior hospitalizations.

**Discussion and Implications:**

Despite calls for minimizing hospital-based deaths and maximizing home-based deaths, older LTC recipients often experience EOL care trajectories that end in death in a nursing home care setting. Our findings point to the value of a structural/institutional life course perspective in informing an understanding of who experiences this and other major EOL care trajectories. In doing so, they also provide direction for policy and practice designed to address inequalities and enhance the quality of EOL care.

**Translational Significance**

Our findings point to the need to focus attention on and ensure access to quality end of life and palliative care services within nursing homes as well as other long-term care settings. The influence of age, gender, and other social, economic, and health factors should be addressed with the goal of ensuring equitable access to appropriate EOL care trajectories.

In concert with population aging and concerns about current and future health care costs, issues around EOL care have attracted increasing attention in recent years, with both the World Health Organization and the United Nations identifying improvements in EOL care as a global public health priority (Broad et al., 2013). In Canada, as in many other countries, the number of adults aged 65 and older is projected to increase significantly over the next two decades and since almost 80% of all deaths take place within this age group (Canadian Institute for Health Information [CIHI], 2008), the number of deaths is projected to increase even more dramatically (e.g., by 65% from 2005 to 2036—see Fowler & Hammer, 2013).

Among the main concerns evident within recent literature on EOL care are over-medicalization and lack of continuity created through numerous care-related transitions, particularly those that involve hospitalization (Coleman & Boult, 2003; Wang et al., 2016). Care transitions are considered particularly problematic for older adults whose experiences with multiple and complex chronic conditions render them especially vulnerable. As a result, they also have a greater potential for adverse outcomes including stress; inappropriate, inconsistent, and poor quality care; declines in health and well-being; and premature mortality (Abarshi et al., 2010; Callahan et al., 2015; Gozalo et al., 2011; Naylor, Kurtzman, & Pauly, 2009; Teno et al., 2013; Wang et al., 2016).

To date, however, research addressing the multiple transitions often embedded within EOL care trajectories in later life is limited. More often, the focus tends to be on specific transitions or the total number of transitions as well as on where people die, with particular attention to hospital deaths and transitions (Gruneir et al., 2007), often during the last few months of life (e.g., Abarshi et al., 2010; Gozalo et al., 2011; Klinkenberg et al., 2005; Teno et al., 2013; Wang et al., 2016). Yet, the long-term nature of the chronic conditions, and thus the protracted nature of the dying process often encountered in later life, suggests the need for a longer-term view. It also calls for recognition of the long-term care system as one that encompasses the diverse forms of care (e.g., health and social services) required to support functioning in daily life. Consequently, we draw on the Organization for Economic Co-operation and Development (OECD) definition of long-term care as “care for people needing support in many facets of living over a prolonged period of time” and provided in-home, institutional, or other settings by home and community-based care as well as residential/nursing home care and other service providers (Colombo, Llena-Nozal, Mercier, & Tjadens, 2011, p. 39).

Given the limited research currently available on the EOL care trajectories of older adults together with the importance of such knowledge for health system improvement and for enhancing the quality of life of older adults nearing the end of their lives, we drew on insights from a structural/institutional life course perspective (Dannefer, 2012; Moen, 2013) and secondary administrative data to address two research questions: First, what are the main EOL care trajectories experienced by older adults transitioning through the formal long-term care (LTC) system? Second, what roles do social location, social and economic resources, and health-related factors play in influencing these trajectories? Addressing these gaps in the literature also provides direction to policy and practice designed to address inequalities and enhance the quality of late life care.

## Background

Concerns about the implications of population aging are accompanied by concerns regarding the increased medicalization of death and dying evident over the course of the 20th century and which saw death increasingly institutionalized in acute hospital care settings (Menec, Lix, Nowicki, & Ekuma, 2007; Wilson et al., 2001). When asked, most people say they would prefer to die at home in familiar surroundings and in the presence of loved ones (CIHI, 2007). Yet, whereas death (and dying) at home was the norm until the early years of the 20th century, from the 1950s through to the 1990s, death became increasingly concentrated in hospital settings, reaching a peak in 1994 when 80% of all Canadian deaths took place in acute care hospitals (Wilson et al., 2001). Since then, however, with evidence regarding people’s preferences for home deaths coinciding with governmental interests in reducing hospital costs (CIHI, 2007), increasing attention has been directed towards relocating death (and dying) back to home or home-like (e.g., hospice) settings. As a result, by 2004, approximately 60% all deaths in Canada took place in hospital settings (CIHI, 2011). Similar reductions in hospital deaths have been reported in other countries as well (e.g., Houttekier, Cohen, Surkyn, & Deliens, 2011; Teno et al., 2013).

Reductions in hospital deaths are frequently attributed to the success of efforts to strengthen EOL care provided in community settings and thereby increase home-based deaths (CIHI, 2011; Gruneir et al., 2007). They also are viewed as evidence of enhanced continuity of care and the success of attempts to bring about reductions in preventable transitions in care, particularly those that involve hospitalization (Naylor et al., 2009). However, in the push to relocate deaths outside of hospital settings, what is less frequently acknowledged is that as hospital deaths have been reduced and home deaths have increased, the number of deaths taking place in nursing home settings has also increased. According to Wilson et al. (2009), from 1994 to 2004, the number of deaths taking place in Canadian nursing homes and similar institutions tripled. After hospitals, LTC facilities now represent the second most common place of death (CIHI, 2007). In the province of British Columbia specifically, in 2003–2004, 53% of deaths took place in hospitals whereas 27% took place in nursing home care facilities and 17% took place in people’s own homes (CIHI, 2008).

Yet, limited research has focused on why it is that some people die at home in the community while others experience one or more transitions that lead to death in a hospital or a nursing home setting. Concerns around the negative impact of transitions, particularly repeated transitions, from home to hospital on the health and mortality of older adults as well as on related concerns with preserving continuity of care have led to research on health care use at the EOL (Menec et al., 2007) as well as the number and type of transitions experienced (e.g., Abarshi et al., 2010; Teno et al., 2013; Van den Block, Deschepper, Bilsen, Van Casteren, & Deliens, 2007) and where people die (Broad et al., 2013; Gruneir et al., 2007; Klinkenberg et al., 2005; Menec, Nowicki, Blandford, & Veselyuk, 2009; Motiwala, Croxford, Guerriere, & Coyte, 2006; Temkin-Greener, Zheng, & Mukamel, 2013; Teno et al., 2013). Findings suggest that health care utilization tends to increase near the EOL (especially the last 3–6 months) and transitions between care settings are common, in Canada (CIHI, 2008) and elsewhere (Abarshi et al., 2010; Klinkenberg et al., 2005; Van den Block et al., 2007).

Studies have also focused on the characteristics of those likely to experience multiple transitions in care as well as of those whose lives end in various settings. Research evidence suggests that older women are less likely to experience one or more care transitions at the EOL (Aaltonen, Forma, Rissanen, Raitanen, & Jylhä, 2010; Aaltonen, Rissanen, Forma, Raitanen, & Jylhä, 2012; Abarshi et al., 2010). In addition, socioeconomic factors (education, income), marital status, informal support, health status (e.g., dementia vs. other causes of death), the availability of palliative care or other health services, and several other factors also appear to influence where people die (Forma, Rissanen, Noro, Raitanen, & Jylhä, 2007; Fowler & Hammer, 2013; Martikainen, Moustgaard, Einiö, & Murphy, 2014; Menec et al., 2007, 2009; Wilson et al., 2009), including the likelihood of transition (e.g., to hospital) at the EOL (Aaltonen et al., 2010; Menec et al., 2007; Motiwala et al., 2006). For example, older (age 85+) women, those with more limited economic (education, income) and social resources (e.g., not married, no children, lack a primary caregiver) appear less likely to die at home in the community than in institutional care settings (Aaltonen et al., 2010; Klinkenberg et al., 2005; Menec et al., 2007; Motiwala et al., 2006; Weitzen, Teno, Fennell, & Mor, 2003).

## The Current Study

The preceding review points to a need for research on care trajectories at the EOL and the factors that influence them. From a life course perspective, it can be argued that it is not only the number of transitions experienced at the very end of life or where the final transition (death) takes place that is important to consider. Instead, insofar as prior transitions are likely to influence subsequent ones, the longer-term trajectories that end in death are also important. Accordingly, we focus on individuals’ transitions through the formal LTC system, including home and community-based care as well as nursing home/residential care settings and other service providers. We draw on a structural/institutional life course perspective (Dannefer, 2012; Moen, 2013) and conceptualize LTC trajectories as being among the multiple social pathways (work, marriage, family life) through which older adults as a social group are likely to pass. These pathways are embedded in and will therefore reflect macro- and meso-level social structural and contextual factors, including the policy contexts within which they are situated, the socially structured inequalities that attend location within particular social groups (e.g., gender, age, geography), the resources and barriers that emanate from these structural forces (e.g., social and economic resources), and the health-related risks that they impose. Accordingly, our objectives are both descriptive and analytical: to examine what the EOL trajectories of older adults who are recipients of publicly provided LTC services look like and to assess the impact of social structural location, associated social and economic resources, and health status factors on these trajectories.

## Methods

### Data Source and Sample

Our analyses drew on secondary administrative data obtained for older adults (aged 65+) who received publicly subsidized long-term care services in one health region in British Columbia. Fraser Health is one of five geographically defined public sector organizations responsible for planning and delivering health services in the province. As an administrative area overseen by the provincial Ministry of Health, Fraser Health is responsible for the delivery of health services (including 12 acute care hospitals, outpatient care, and surgery centre, 7,760 residential care beds, mental health care, public health, and home and community care services) to a total population of approximately 1.8 million people (36% of the provincial population) who live in the region. These data sources included individually linked client demographic and service history files; hospital separations data files (i.e., the *Discharge Abstracts Database*—see https://www.cihi.ca/en/dad_multi-year_en.pdf); and client assessment data (including the *Resident Assessment Instruments - Minimum Data Sets* for *Residential Care* [RAI-MDS 2.0] and *Home/Community Care* [RAI-HC], Canadian versions). These latter data sets provide client assessment data using standardized, comprehensive, and validated instruments developed by interRai, an international research collaborative (Hirdes, Mitchell, Maxwell, & White, 2011). Client assessments are completed at regular intervals (upon admission to care and every 3 [RAI-MDS] to 12 [RAI-HC] months thereafter), or when there is a substantial change in health status. Clients aged 65 and older as of January 1, 2008 who had at least one active LTC service record from January 1, 2008 through December 31, 2011, and who died by March 31, 2012 were included in the study. The period of death was selected to coincide with the time periods used in the hospital data required for establishing the location of death. Overall, 13,395 decedents were identified representing approximately 34% of all deaths evident among adults aged 65 and older in the region during this period. Of these, 1,579 were excluded from the final analysis, primarily because they did not have any formal assessment data in their files.

It should be noted that in Canada, medically necessary physician and hospital services are universally insured publicly funded services. In contrast, LTC services offer a mix of universal and means-tested benefits that are highly variable across provinces. Across the country, LTC is delivered by governmental as well as for-profit and not-for-profit providers, on both a publicly subsidized and private-pay basis. In British Columbia, LTC includes home and community-based services such as direct care (home nursing, occupational therapy, physiotherapy, social work, nutritional services) and home support services (assistance with mobility, nutrition, lifts/transfers, bathing, grooming/toileting, and cueing). They are supplemented by assisted living, nursing home/residential care (RC), and other services. For those who are eligible (based on citizenship, residency, age, and assessed need for care – post-hospital, due to terminal illness, or due to an inability to function independently due to chronic health-related problems), there is no cost for direct care services delivered by public sector employees whereas home support services require that care recipients, excluding those with low incomes, pay a daily rate based on income for services delivered by private agencies. Assistance with housekeeping and other instrumental activities of daily living (ADL) is generally not available through the public system. Assisted living and RC are available on both a publicly subsidized and private-pay basis in fully private, fully public, and mixed buildings, with recipients once again assessed a monthly rate determined according to income and/or assets.

### Measures

To measure EOL care trajectories, we relied on service records indicating the start and end dates for the receipt of publicly subsidized home and community care (HCC) services (including home support, direct care services, day programs, respite/convalescent care, assisted living, other services) as well as RC services. These data along with the date and location of death (drawn first from hospital records to identify those who died in hospital and from service record data to identify those who died outside hospital) were used to generate distinct EOL care trajectories. Although we did not have data on the location of death for those who died outside of hospital, our assumption was that those who died a non-hospital death usually died in their place of residence (i.e., either at home or in the nursing home).

Framed by a structural/institutional life course perspective, social location, social and economic resources, and health factors were also included in the analyses (Dannefer, 2012; Moen, 2013).

#### Social location

Age was a continuous measure, assessed in years. Gender was coded as a dummy variable. Geography was determined using postal code information geocoded into one of three categories: rural, suburban, and urban core.

#### Social and economic resources

Whether the care recipient was married, lived alone (at the last assessment prior to death for HCC residents and prior to RC entry for RC residents), and had a legal guardian responsible for decision-making regarding their care were included to measure access to social resources. Economic covariates included low income status and responsibility for payment. Low income status was determined based on whether or not the care recipient received the Guaranteed Income Supplement (GIS), a federal income supplement paid to older adults with poverty-level incomes. A variable assessing missing data on the income variable was also included to examine its implications. To assess responsibility for payment, we also dichotomized care recipients whose care included private payment versus those whose care did not.

#### Health factors

Health status was assessed using several indicators. Physical health was measured using the total number of chronic conditions (from 18 conditions including stroke, hypertension, arthritis, cancer, diabetes, etc.). In addition, activity limitations were assessed using the Activities of Daily Living (ADL) Self-Performance Hierarchy Scale (Morris, Fries, & Morris, 1999), which takes into account both the level of dependence (seven categories ranging from independent to totally dependent) and specific activities (personal hygiene, toileting, locomotion, and eating). Scores ranged from 0 to 6, with higher scores indicating greater need for assistance with ADLs. Risk of falls was based on an assessment of the client as being at: (0) no/low risk, (1) medium risk, or (2) high risk of future falls. Finally, health instability was assessed using the Changes in Health, End-Stage Disease, Signs, and Symptoms Scale (CHESS), a pre-validated measure of illness and disability often used to predict whether a client is at the EOL (Hirdes, Frijters, & Teare, 2003). Scores ranged from 0 to 5, with higher scores indicating greater instability.

To assess mental health, the Depression Rating Scale (Burrows, Morris, Simon, Hirdes, & Phillips, 2000) was included. Based on seven items, possible scores ranged from 0 to 14, with higher values indicating more numerous and/or frequent symptoms. A log transformation was implemented to adjust for skewness. Cognitive functioning was assessed using the MDS Cognitive Performance Scale (Morris et al., 1994). Possible scores ranged from 0 (intact) to 6 (very severe impairment). Finally problematic behavior was assessed using a dichotomous measure reflecting whether (coded as 1) or not (coded as 0) clients exhibited one or more behaviors such as: wandering, verbal abuse, physical abuse, disruptive behavior, or resisting care.

We also included variables capturing changes in key health status indicators (ADL, cognitive performance) over time. Other health status indicators showed minimal change and thus were not included. Individual annual rates of change were computed using measures of these indicators across all time points. For both ADL and cognitive functioning, final values distinguished those experiencing greater decline (i.e., with change scores at or above the median, coded as 1) from those experiencing little change or even improvement (coded as 0).

Finally, we also included a measure of the number of hospitalizations experienced in the 90 days prior to death (excluding the final hospitalization for those who died in hospital), comparing those with no, one, and two or more hospitalizations during this period.

### Statistical Models

Multinomial logistic regression analyses were used to assess the impact of various covariates on the eight EOL care trajectories identified above. Reflecting the structural/institutional life course perspective elaborated above, predictor variables were entered sequentially in a series of nested models, with social location variables entered in model 1, social and economic resources in model 2, baseline health status variables in model 3, changes in health status in model 4, and recent hospitalizations in model 5. Here we report on the results obtained in the final model (model 5).

## Results

### Descriptive Statistics

**Figure 1.**
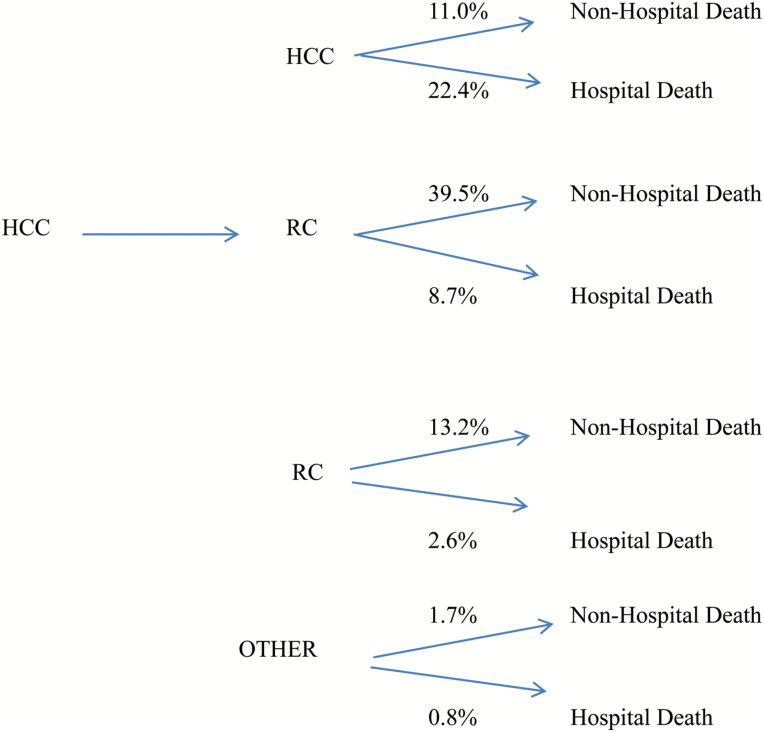
End-of-life care trajectories.

Table 1 provides descriptive statistics for all variables used in our analyses. Just over one-third (34.5%) of those in our client sample died in hospital; the remainder (65.4%) died outside hospital. Among those who died while living at home and receiving HCC, the most common scenario, accounting for about two-thirds of all deaths, was to die in hospital. Among those who died while living in RC settings, in contrast, the vast majority (over 80%) died outside hospital, most likely in the RC setting in which they lived. Overall, eight EOL trajectories through the LTC system were identified (see Figure 1): (1) HCC to non-hospital death (*n* = 1,304; 11.0%); (2) HCC to hospital death (*n* = 2,645; 22.4%); (3) HCC to RC to non-hospital death (*n* = 4,669; 39.5%); (4) HCC to RC to hospital death (*n* = 1,029; 8.7%); (5) RC to non-hospital death (*n* = 1,560; 13.2%); (6) RC to hospital death (*n* = 309; 2.6%); (7) Other (primarily alternating HCC and RC) to non-hospital death (*n* = 200; 1.7%); and (8) Other (primarily alternating HCC and RC) to hospital death (*n* = 100; 0.8%). Unlike the first six trajectories, the latter two are generally non-linear and characterized by repeated use of HCC and/or RC services (e.g., HCC to RC to HCC, RC to HCC to RC, etc.). In addition, a few also transitioned from RC to HCC. Overall, the single most frequently observed trajectories involved transitions from HCC to RC to non-hospital death or from HCC to non-hospital death. As well, most trajectories were relatively straight forward and linear: very few (2.5%) involved moves from RC to HCC or the repeated use of HCC and RC services.

**Table 1.** Descriptive Statistics for Variables Used in the Analyses

	Overall	HCC > death not in hospital	HCC > death in hospital	HCC > RC > death not in hospital	HCC > RC > death in hospital	RC > death not in hospital	RC > death in hospital	Other > death not in hospital	Other > death in hospital
Measure	x̅ (*SD*) or %	x̅ (*SD*) or %	x̅ (*SD*) or %	x̅ (*SD*) or %	x̅ (*SD*) or %	x̅ (*SD*) or %	x̅ (*SD*) or %	x̅ (*SD*) or %	x̅ (*SD*) or %
Age at death	86.5 (7.2)	85.2 (7.7)	85.5 (7.3)	87.5 (7.0)	85.7 (6.9)	86.8 (7.0)	85.0 (6.9)	87.2 (7.8)	85.9 (7.3)
Gender									
Male	38.0	37.7	40.9	35.0	41.4	40.2	46.8	25.0	28.0
Female	62.0	62.3	59.1	65.0	58.6	59.8	53.2	75.0	72.0
Location of residence									
Rural	12.7	14.8	15.8	13.3	8.4	8.7	4.2	12.5	7.0
Suburban	33.1	34.2	33.9	34.2	32.2	30.4	27.8	28.0	25.0
Urban	54.2	51.2	50.5	52.5	60.1	60.7	68.4	59.5	68.0
Marital status									
Married	31.8	33.5	33.4	31.2	32.9	30.8	30.9	22.2	26.0
Widowed	56.1	53.5	54.5	58.1	53.5	56.4	50.5	66.0	62.0
Other	12.0	13.0	12.1	10.7	13.6	12.8	18.6	11.9	12.0
Lived alone	24.9	37.8	40.5	17.0	23.6	13.4	23.6	17.0	27.0
Legal guardian	40.8	49.5	47.4	37.1	40.9	35.1	33.7	34.0	44.0
Receipt of low income supplement									
Yes	39.5	48.7	53.0	34.9	40.4	22.9	32.4	38.0	50.0
No	47.9	46.2	42.5	48.2	50.8	56.0	56.0	34.0	39.0
Unknown	12.6	5.1	4.5	16.8	8.7	21.1	11.7	28.0	11.0
Private pay	18.8	24.1	24.8	15.8	16.6	15.5	18.4	16.0	19.0
Number of chronic conditions	2.3 (2.2)	3.6 (1.9)	3.8 (1.9)	1.6 (2.1)	2.2 (2.3)	1.3 (1.7)	1.8 (2.0)	1.8 (2.2)	3.4 (2.6)
ADL Self- Performance Scale	2.7 (1.9)	1.7 (1.8)	1.4 (1.7)	3.3 (1.8)	2.7 (1.7)	3.5 (1.8)	2.8 (1.7)	3.5 (1.8)	2.5 (1.9)
Falls risk									
No/minimum risk	62.6	57.5	56.1	65.9	62.2	68.1	58.3	71.5	61.0
Medium risk	19.6	23.2	22.8	17.3	20.8	18.3	20.1	13.0	19.0
High risk	17.8	19.3	21.2	16.8	17.0	13.7	21.7	16.5	20.0
Medical frailty (CHESS)	1.6 (1.2)	1.4 (1.1)	1.5 (1.1)	1.7 (1.2)	1.5 (1.2)	1.6 (1.2)	1.4 (1.2)	1.6 (1.2)	1.5 (1.1)
Depression Rating Scale	1.4 (2.2)	1.3 (2.2)	1.2 (2.1)	1.5 (2.3)	1.5 (2.3)	1.3 (2.2)	1.5 (2.2)	1.5 (2.3)	0.8 (1.5)
CPS	2.6 (1.8)	1.7 (1.6)	1.6 (1.4)	3.2 (1.7)	2.5 (1.5)	3.4 (1.7)	2.6 (1.5)	2.8 (1.8)	2.2 (1.7)
Behavioral concerns	14.2	9.0	9.6	16.8	15.2	17.0	19.7	13.0	14.0
Decline in ADL	49.9	45.1	43.6	60.8	56.5	34.7	24.6	44.0	31.0
Decline in cognitive performance	49.9	46.9	42.2	57.6	51.8	44.1	45.6	43.5	28.0
Hospitalizations in 90 days pre death									
0	65.5	44.2	61.7	71.4	66.1	71.1	64.1	72.0	69.0
1	24.6	37.0	24.5	22.0	25.0	22.1	26.9	24.0	18.0
2+	9.9	18.8	13.8	6.6	8.9	6.9	9.1	4.0	13.0
*N*	11,816	1,304	2,645	4,669	1,029	1,560	309	200	100

*Note:* ADL = activities of daily living; CHESS = Changes in Health, End-Stage Disease, Signs, and Symptoms Scale; CPS = Cognitive Performance Scale; HCC = home and community care; RC = residential care. Bivariate significance tests were conducted using chi square or analysis of variance as appropriate. All *p* values were significant at *p* <0.001.

The median number of years that clients within the first six trajectories spent in LTC ranged from 1.46 to 4.21 years. The overall duration of care was the shortest among those who transitioned directly from RC to hospital (1.46 years) or non-hospital death (1.88 years). It was somewhat longer among those who transitioned from HCC to hospital (3.08 years) or to non-hospital death (3.20 years). Finally, it was longest among those who transitioned from HCC to RC to hospital (3.64 years) or to non-hospital death (4.21 years). In general, clients spent more time receiving HCC than RC services.

### Covariate Analyses

Table 2 reports findings regarding the influence of social location, social and economic resources, and health factors on these EOL care trajectories (final model).

**Table 2.** Multinomial Logistic Regression of EOL Trajectory With HCC to Non-hospital Death as Reference, 2008–2011, Final Model

	HCC > hospital death	HCC > RC > non-hospital death	HCC > RC > hospital death	RC > non- hospital death	RC > hospital death	Other > non- hospital death	Other > hosp death
	OR	OR	OR	OR	OR	OR	OR
Intercept (est.)	0.583	−3.432***	−1.268*	−3.214***	−1.218	−4.629***	−2.019
Social location							
Age at death***	1.004	1.047***	1.012^+^	1.035***	1.002	1.025*	0.998
Gender (male = 1)***	1.241**	1.032	1.232*	1.297**	1.465**	0.729	0.720
Location of residence (urban = ref.)***							
Rural	1.152	0.740**	0.421***	0.408***	0.174***	0.635^+^	0.369*
Suburban	1.039	1.246**	0.975	0.989	0.778^+^	0.865	0.605*
Social and economic resources							
Marital status (married = ref.)***							
Widowed	0.971	1.515***	1.506***	1.924***	1.874***	2.077*	1.668^+^
Other	0.839	1.652***	1.704***	2.412***	2.792***	2.044***	1.606
Lived alone***	1.085	0.789**	0.827^+^	0.697**	0.939	0.666^+^	0.628^+^
Legal guardian	0.906	0.962	0.945	1.023	0.710*	0.889	0.883
Receipt of low income supplement (no = ref.)***							
Yes	1.220**	0.681***	0.756**	0.391***	0.544***	0.961	1.096
Unknown	0.900	1.618***	1.021	1.537**	0.992	3.580***	0.647
Private pay	1.091	0.951	0.799^+^	1.036	1.080	1.090	0.863
Health factors							
Number of chronic conditions***	1.032^+^	0.684***	0.778***	0.620***	0.666***	0.759***	1.002
ADL Self- Performance Scale***	0.928**	1.396***	1.264***	1.529***	1.372***	1.615***	1.275***
Falls risk (none/minimal = ref.)*							
Medium	1.007	0.947	1.027	1.035	1.147	0.690	0.855
High	1.119	1.299**	1.083	1.122	1.633**	1.167	1.189
Medical frailty (CHESS)***	1.074*	1.184***	1.022	1.174***	0.999	1.150*	1.064
Depression Rating Scale (log)***	0.979	1.242***	1.297***	1.125^+^	1.289**	1.228^+^	0.692*
Cognitive Performance Scale***	0.924**	1.265***	1.127***	1.392***	1.143**	1.124*	1.135
Behavioral concerns***	1.185	1.620***	1.413*	1.738***	2.186***	1.308	1.585
Decline in ADL***	1.023	1.286***	1.318**	0.392***	0.285***	0.670*	0.615*
Decline in cognitive performance***	0.852*	0.818**	0.846^+^	0.587***	0.981	0.628**	0.423***
Number of hospitals in last 90 days***							
One	0.456***	0.516***	0.524***	0.509***	0.556***	0.539***	0.319***
Two+	0.485***	0.395***	0.451***	0.442***	0.502**	0.230***	0.545^+^
Likelihood ratio	6251.718***						
df	161						
*N*	11,816						

*Note:* ADL = activities of daily living; CHESS = Changes in Health, End-Stage Disease, Signs, and Symptoms Scale; EOL = end of life; HCC = home and community-based care; OR = odds ratio; RC = residential/nursing home care.

***p < .001. **p < .01; *p < .05; ^+^*p* < .10 (two-tailed test).

### Social Location

With regard to social location, the findings reveal that being older increased the likelihood of experiencing EOL trajectories that involved RC transitions and non-hospital deaths (i.e., HCC to RC to non-hospital death, RC to non-hospital death, or alternating use of both HCC and RC to non-hospital death). On the other hand, with age and other factors controlled for, older men were more likely than older women to die in hospital settings regardless of whether their deaths occurred in conjunction with HCC or RC. However, being male also increased the likelihood of a RC to non-hospital death compared to a HCC to non-hospital death. Finally, compared to urban-dwelling respondents, those who lived in rural areas were less likely to experience EOL trajectories that involved RC transitions (i.e., transitions from HCC to RC to hospital or non-hospital death; RC to hospital or non-hospital death) compared to HCC to non-hospital deaths. However, no differences emerged in their likelihood of experiencing a HCC to hospital rather than a HCC to non-hospital death.

### Social and Economic Resources

Social and economic resources also had a significant impact on EOL care trajectories. Older adults who were not married, whether widowed or divorced/separated, were significantly more likely than those who were married (i.e., the reference category) to experience EOL care trajectories involving RC (including hospital and non-hospital death) rather than HCC (with death either in or outside hospital). They also were more likely to experience alternating use of HCC and RC followed by a non-hospital death. With the impact of marital status accounted for, those who lived alone prior to death (HCC) or institutionalization (RC residents) were found to be less likely to undergo trajectories involving RC and non-hospital death (i.e., HCC to RC to non-hospital death, RC to non-hospital death) than a HCC to non-hospital death. Having a legal guardian had limited impact, somewhat reducing the likelihood of a RC to hospital death compared to a HCC to non-hospital death. Receiving a low income subsidy also reduced the likelihood of experiencing EOL care trajectories that included moves from RC to death (i.e., HCC to RC to death, RC to death), regardless of the location of death, compared to moves from HCC to non-hospital death. Yet, it also increased the likelihood of experiencing a HCC to hospital death trajectory rather than a HCC to non-hospital death trajectory. A low income subsidy had little impact on the likelihood of experiencing patterns of alternating HCC and RC use, whether it led to a hospital or non-hospital death. In general, private payment for care had limited impact, although there was some indication that those who were required to pay for some or all of their care privately were also less likely to experience trajectories involving moves from HC to RC to hospital death than they were to experience transitions from HCC to non-hospital death.

### Health Factors

Physical and mental health status indicators also emerged as significant. Interestingly, those with more chronic conditions were less likely to experience trajectories involving RC, including those that ended with death either in or outside the hospital setting, than they were to experience a HCC to non-hospital death trajectory. Conversely, those with greater ADL impairments were more likely to experience trajectories involving RC, regardless of whether they ended with death in or outside the hospital setting, than they were to experience a HCC to non-hospital death trajectory. They were also less likely to experience a HCC to hospital death than they were to experience a HCC to non-hospital death. A high risk of falls increased the likelihood of experiencing a HCC to RC to non-hospital death as well as a RC to hospital death compared to the reference. Finally, individuals with higher levels of medical frailty (i.e., higher CHESS scores) were more likely to experience sequential as well as alternating HCC to RC to non-hospital death trajectories as well as direct RC to non-hospital death trajectories relative to HCC to non-hospital deaths. They were also more likely to experience HCC to hospital deaths than HCC to non-hospital deaths.

With regard to mental health, our analyses revealed that those with higher levels of depression were more likely to experience EOL trajectories involving moves from RC to death, whether preceded by HCC or not and whether followed by a hospital death or not, than they were to experience HCC to non-hospital or hospital death trajectories. Conversely, those with higher levels of depression appeared significantly less likely to experience trajectories characterized by alternating HCC and RC followed by hospital death than they were to experience a HCC to non-hospital death trajectory. Those assessed as having more severe cognitive impairment and as demonstrating one or more problematic behaviors also had a greater likelihood of experiencing most of the trajectories involving RC compared to HCC to non-hospital care, with the former also reducing somewhat the likelihood of experiencing a HCC to hospital EOL trajectory compared to a HCC to non-hospital death. In contrast, cognitive impairment did not differentiate those in alternating HCC and RC to hospital death trajectories from the reference category. Nor were problematic behaviors significant in differentiating those involved in alternating HCC and RC to either hospital or non-hospital death trajectories from the reference category.

Taking these health status factors into account, our findings also revealed that those who experienced greater declines in ADL functioning over time were more likely to experience EOL care trajectories involving moves from HCC to RC to death (either within or outside hospital) but less likely to experience moves from RC to death (hospital or non-hospital) or to alternate back and forth between HCC and RC before death (hospital or non-hospital) than they were to experience HCC to non-hospital death trajectories. Declines in cognitive performance also reduced the likelihood of experiencing most other EOL trajectories—including HCC to hospital death, HCC to RC to non-hospital death, RC to non-hospital death, and alternating HCC and RC followed by either hospital or non-hospital death—compared to HCC to non-hospital death.

Finally, those experiencing one or more prior hospitalizations in the last 3 months of their lives were less likely to experience all EOL care trajectories involving either a hospital or RC death (including HCC to hospital death, HCC to RC to hospital and non-hospital deaths, RC to hospital and non-hospital deaths, and alternating HCC and RC to hospital and non-hospital death) than a HCC to non-hospital death.

## Discussion

This study drew on a structural/institutional life course perspective and longitudinal data to: (1) identify the main EOL trajectories experienced by older adults transitioning through the publicly supported LTC system and to (2) assess the roles of social location, social and economic resources, and health-related factors in influencing them.

With regard to the first objective, our findings revealed that, in general, LTC clients’ EOL trajectories were generally linear and straight forward: very few (2.5%) experienced trajectories that involved transitions from RC to HCC or more than a single transition between HCC and RC (e.g., HCC to RC to HCC to death). Instead, the vast majority of older clients began their trajectories with the receipt of HCC services. Just over one-third (33.4%) then transitioned directly from HCC to death, most often in a hospital setting. In addition, almost one-half (48.2%) transitioned from HCC to RC to death, typically in a non-hospital setting. Although a minority began their trajectories in RC, most (66.6%) older clients’ trajectories included RC and the vast majority (81.7%) of those whose trajectories included RC subsequently experienced a non-hospital death. Findings indicating that just over 18% of RC residents died in hospital are comparable to those reported in another Canadian study (Menec et al., 2009) and slightly lower than figures reported for the United States (20.4% from 2003 to 2007—see Temkin-Greener et al., 2013).

Overall, the fact that almost two-thirds (65.4%) of those in our sample died outside of hospital settings and that those most likely to do so were those receiving RC rather than HCC services suggest that concerns regarding a highly medicalized and technological versus a comfort-focused home-based death may be more relevant to recipients of home-based LTC services than to older residents of nursing home care settings. This is further supported by findings indicating that over 70% of those whose LTC trajectories ended with a nursing home death had no hospitalizations in the 3 months prior to death compared to only 44% of those who died at home.

As well, findings indicating that most RC residents who died did not die in hospital and did not experience any hospitalizations in the months prior to their deaths could be considered consistent with recent economic, policy, and quality of care objectives (emphasizing the need to minimize unnecessary and often problematic transitions for older adults, especially those with dementia or multiple chronic conditions and complex care regimens for whom such transitions often pose challenges to continuity and quality of care—e.g., Naylor et al., 2004). On the other hand, not all transitions should be considered unnecessary and specifying an appropriate rate appears problematic (Sivananthan & McGrail, 2016). Also, concerns remain regarding the quality of nursing home EOL care (CIHI, 2013; Mitchell, Teno, Miller, & Mor, 2005). When asked, few people identify nursing homes as places they wish to receive care or where they wish to end their lives (Wilson et al., 2009). Further, it has been noted that nursing homes in Canada provide nursing and personal care but do not provide palliative care services and “do not emphasize that they provide care to dying people” (Wilson et al., 2009, p. 1756). For example, it has been reported that only 18.3% of those who died in RC facilities in British Columbia in 2003/2004 received some form of palliative care (CIHI, 2008).

Our second objective was to assess the roles of social location, social and economic resources, and health-related factors in influencing the care trajectories experienced by older clients at the EOL. As noted, a structural/institutional LCP considers age as well as gender and geography as indicators of location within a stratified (unequal) social structure. Our findings revealed considerable empirical support for the importance of such factors in influencing EOL care trajectories. For example, older clients were more likely to experience trajectories that included RC transitions and also, to experience EOL trajectories that ended outside of hospital. This was evident regardless of the inclusion of social and economic resources, health status/needs, or recent hospitalizations in the model, suggesting that the impact of age was not attributable to such factors. Similar findings have been reported by others (e.g., Menec et al., 2007) and support the view that old age itself is a key determinant of EOL care (Forma et al., 2007). There are several possible explanations. First, there is the possibility that within the public LTC system, those in advanced old age are funneled into institutional (nursing home) rather than community-based LTC settings (Penning, Cloutier, Nuernberger, MacDonald, & Taylor, 2016) and, in the process, into a trajectory that will see their lives come to an end within the nursing home itself. In addition, as noted by Forma et al. (2007, p. 152), “regardless of need, older age groups may have fewer admissions to inpatient hospital care than younger ones because they are less likely admitted and less intensive care is given for them” (see Levinsky et al., 2001). This also suggests a rationing of hospital EOL care away from older adults in RC settings.

EOL care trajectories also differed depending on gender and geography. Consistent with previously reported findings (e.g., Forma et al., 2007), older men were generally more likely than older women to experience trajectories that concluded with death in a hospital setting. Although women are often reported to use more health services than men, this appears to vary with the type of service involved, with gender inequities in the direction of greater use by men reported in access to acute hospital care (e.g., Dunlop, Manheim, Song, & Chang, 2002; Song, Chang, Manheim, & Dunlop, 2007). Forma et al. (2007) speculate that this may reflect the fact that older women spend more time in RC institutions than men due to their greater likelihood of living alone, reduced access to informal care, and more severe disabilities. However, we were able to control for the impact of living arrangements, marital status, as well as several health status factors (and economic resources), albeit not informal care, with the result that the impact of gender remained. Similar findings are reported by Song et al. (2007). With regard to geography, compared to urban-dwelling respondents, those who lived in rural areas were less likely to experience EOL trajectories that involved RC transitions compared to HCC transitions (including hospital and non-hospital deaths).

Findings indicating that as was the case with regard to age, the impact of gender and geography on EOL care trajectories remained significant despite the inclusion of social and economic resources or health status indicators in our models suggests that there is in fact something else about these indicators of social structural location that leads to different EOL care trajectories. One possibility, of course, is that these findings reflect our failure to include the most relevant indicators of such factors (e.g., availability of informal caregivers, specific cause of death) in our models. Another possibility, however, is that they reflect the impact of macro- and/or meso-level contextual factors that lead to the differential social structuring of age, gender, and rural versus urban-related LTC and EOL trajectories. This includes health care policy and service delivery contexts that may consider older women to be less capable of functioning independently in the community setting, that may regard older men as being more appropriate candidates for acute hospital and medical care, and that provide for differential access to hospital, nursing home, and community-based resources in rural and urban areas.

Importantly, social and economic resources also influenced LTC trajectories, including their end-points. The finding that those who were not married were more likely to experience trajectories involving RC than HCC, regardless of whether these ended in death in or outside hospital, appears to support prior research attesting to the importance of close family relationships for the ability to live in the community when health declines (e.g., Gaugler, Duval, Anderson, & Kane, 2007). Controlling for marital status, it was those who were living with others who were more likely to experience EOL care trajectories involving RC, particularly those involving non-hospital death. On the one hand, this points to the possibility that those with greater disability were more likely to live with others and subsequently, to enter RC settings when their care needs increased. However, it may also speak to the role of family members in providing guidance to RC facilities with regard to decision-making around EOL care (e.g., providing support for decisions to pursue EOL care that does not involve hospitalization).

Findings pointing to the importance of economic resources in influencing EOL care trajectories must be interpreted within the context of governmental LTC funding policies. In this study, low income levels reduced the likelihood of older adults experiencing EOL care trajectories that included transition from RC to death (either in or outside hospital) compared to HCC to death (in or out of hospital). In British Columbia, publicly subsidized HCC is primarily targeted to those with low incomes. RC services, in contrast, are accessible to individuals at all income levels with subsidy levels dependent on income and assets. Our findings also revealed an association between low incomes and an increased likelihood of experiencing a HCC to hospital death trajectory rather than a HCC to non-hospital death trajectory. We are not sure why this was the case but speculate that it may reflect the greater involvement of HCC staff in the care trajectories of those with the lowest income levels.

Finally, in terms of health status, we found that greater cognitive and functional impairment increased the likelihood of experiencing EOL trajectories that included RC care (and a hospital or non-hospital death) and reduced the likelihood of experiencing a HCC to hospital death as opposed to a HCC non-hospital death trajectory. Similarly, higher levels of depression and exhibiting behaviors labeled as problematic were associated with the likelihood of experiencing an EOL care trajectory that included RC (and a hospital or non-hospital death) whereas greater medical instability increased the likelihood of RC accompanied by non-hospital death. Conversely, having more chronic conditions increased the likelihood of experiencing a HCC to hospital death rather than a HCC to non-hospital death, but decreased the likelihood of experiencing EOL trajectories that involved RC (including those involving HCC as well as RC and hospital and non-hospital deaths). These findings not only attest to the importance of health factors in influencing EOL care trajectories but also link functional, mental health (cognitive impairment, depression), and behavioral concerns to EOL trajectories centered around RC.

Overall, our findings indicate several relatively distinct patterns with regard to various EOL care trajectories. For example, recipients of HCC EOL trajectories were likely to be rural residents, slightly younger than those who died in RC settings, to have low income levels, to be married, and to have more chronic conditions but lower levels of ADL and cognitive impairment, depression, and problematic behaviors. Among older HCC recipients, those most likely to experience a hospital death were men, those with lower incomes and poorer physical health status (based on chronic conditions and CHESS scores) but better ADL and cognitive functioning, and no hospitalizations within the 90 days prior to death. Conversely, those more likely to experience EOL trajectories involving RC (whether or not these also involved HCC and whether or not they ended in a hospital or non-hospital death) were more likely to be urban residents, unmarried, not poor, and to have fewer chronic conditions but greater ADL and cognitive impairment as well as higher levels of depression and problematic behavior. There were relatively few differences evident when comparing the determinants of EOL care trajectories among RC residents who entered RC care directly to those who moved from HCC to RC. As well, relatively few differences emerged when comparing RC residents who experienced a hospital or non-hospital death. RC residents who experienced a non-hospital death—most likely a death inside the RC facility—tended to be older, female, to be less likely to have lived alone prior to institutionalization, to have unknown income levels, and to have higher medical instability.

Several methodological issues should be considered when interpreting these findings. First, we focused only on the EOL trajectories evident among older adults transitioning through the publicly subsidized Canadian long-term care system in one specific health region. Thus, our results do not speak to the EOL trajectories experienced by the majority of older adults who do not make use of such services. Experiences will necessarily differ among those relying exclusively on privately provided (paid, unpaid) services or living in locales with different policies and service options. In addition, we focused on LTC trajectories that ended in death over a recent 4-year period for which there were complete assessment and client data. These trajectories might look different if assessed over a different period. As well, hospitalizations were included as a predictor and as an outcome (place of death) but not as a component of the trajectories themselves. Finally, although we included changes in functional impairment and cognitive status in our analyses, some factors could be assessed at baseline only (e.g., marital status, income) or were unavailable for all or part of the sample (e.g., informal caregivers). Further, we did not focus on the role of specific diseases or causes of death within our analyses.

These limitations point to a need for further research. Yet, our findings have implications for theory and research as well as for policy and the delivery of EOL and palliative care services. They point to the presence of several distinct but overlapping LTC trajectories at the EOL and also point to the utility of a structural/institutional LCP for understanding these trajectories and the factors that influence them at the individual level. However, they also suggest a need to address the impact of intersecting inequalities (e.g., age, gender, geography) as well as to focus attention further upstream on the role of macro- and meso-level factors. These include whether and how health care policy and service delivery contexts differentially structure EOL and LTC trajectories on the basis of social location (e.g., through policies and procedures that direct older women, those with dementia, and so forth to RC facilities and non-hospital deaths while directing rural residents to HCC and older men to hospital deaths) as well as access to social, economic, and health resources. Findings indicating that advanced age and other factors also structure opportunities for ensuring quality of care while dying suggest avenues for enhancing equitable access to EOL care. Finally, evidence pointing to the fact that most of the older adults we studied died outside of hospital settings, typically in RC, also points to the need to ensure access to quality EOL and palliative care services within such settings. As the population ages, the number of deaths taking place within these places can be expected to increase substantially, making this an important and time-sensitive goal.

## Funding

This work was supported by grants from the Canadian Institutes for Health Research (CIHR): Partnerships in Health System Improvement (PHSI) Grant Program; the Michael Smith Foundation for Health Research (MSFHR) (to M. Penning, D. Cloutier, K. Nuernberger, and D. Taylor, 2012–2016, grant number 122184); and a University of Victoria Internal Research/Creative Project Grant (IRCPG) (to M. Penning, 2016–2017).

## Conflict of Interest

None reported.
